# Role of Cell-Free DNA and Deoxyribonucleases in Tumor Progression

**DOI:** 10.3390/ijms222212246

**Published:** 2021-11-12

**Authors:** Ludmila Alekseeva, Nadezhda Mironova

**Affiliations:** Institute of Chemical Biology and Fundamental Medicine, SB RAS, Lavrentiev Ave., 8, 630090 Novosibirsk, Russia; mila_alex@ngs.ru

**Keywords:** circulating cell-free DNA, neutrophil extracellular traps (NETs), tumor and metastasis development, DNases

## Abstract

Many studies have reported an increase in the level of circulating cell-free DNA (cfDNA) in the blood of patients with cancer. cfDNA mainly comes from tumor cells and, therefore, carries features of its genomic profile. Moreover, tumor-derived cfDNA can act like oncoviruses, entering the cells of vulnerable organs, transforming them and forming metastatic nodes. Another source of cfDNA is immune cells, including neutrophils that generate neutrophil extracellular traps (NETs). Despite the potential eliminative effect of NETs on tumors, in some cases, their excessive generation provokes tumor growth as well as invasion. Considering both possible pathological contributions of cfDNA, as an agent of oncotransformation and the main component of NETs, the study of deoxyribonucleases (DNases) as anticancer and antimetastatic agents is important and promising. This review considers the pathological role of cfDNA in cancer development and the role of DNases as agents to prevent and/or prohibit tumor progression and the development of metastases.

## 1. Introduction

The presence of cell-free DNA (cfDNA) in blood plasma was first described by Mandel and Métais in 1948 [1]. However, researchers did not pay much attention to this work until 1977, when Stroun and Anker described in detail the circulating cfDNA of body fluids in some higher organisms [2]. Today, a large amount of data has been accumulated on circulating cfDNA in the blood of mammals, but the functions of this cfDNA are still not well understood. In the clinical setting, attempts have been made to use cfDNA as a liquid biopsy for the early diagnosis of various diseases or for prenatal diagnostics [3,4]. Despite the success achieved in the search for new markers of tumor progression presented in the pool of cfDNA, the insufficient efficiency of the methods and frequent false positive results markedly limit the application of cfDNA-based liquid biopsy in clinical settings [5]. On the other hand, an increased level of microsatellite fragments, tandem repeats and mobile genetic elements (MGE) is usually detected in the blood-derived cfDNA pool at the early stages of the development of both experimental tumors and various tumors in patients [6,7,8,9]. Therefore, these sequences are considered promising prognostic and diagnostic markers in oncology.

A thorough study of cfDNA characteristics raises the question of the possible role of cfDNA in carcinogenesis. According to the genometastatic hypothesis, ‘metastases can occur by transfection of vulnerable cells located in the target organs with tumor-specific DNA from cells of the primary tumor circulating in the blood plasma’ [10]. This hypothesis has been accepted by other authors as a model that could explain the inconsistencies in experimental data regarding metastasis [11]. In several studies, researchers have shown that tumor-derived cfDNA, which includes fragments of oncogenes, can behave like oncoviruses, which opens up an alternative pathway for metastasis spreading [12,13,14]. The discovery of DNA-containing microvesicles (MVs) and the data demonstrating horizontal DNA transfer between various cells in vitro and organisms have strengthened this hypothesis [15].

The discovery of the phenomenon of neutrophil extracellular traps (NETs) has changed the existing concepts of immunology [16]. In 2003, it was discovered that neutrophils and other granulocytes can release their own DNA into the extracellular space and decorating it with the contents of the granules with the formation of special NETs, which are capable of catching and killing pathogens [17]. It has been repeatedly noted that in many cases, tumor development and metastasis are accompanied by excessive NET formation, which not only enhances adhesion and invasion, but in some cases also allows the tumor to avoid immune surveillance [18].

Taking into account the possible pathological role of cfDNA in carcinogenesis, the development of new antitumor and antimetastatic drugs based on deoxyribonucleases (DNases) is currently underway. In this review, we focus on the role of tumor-derived cfDNA in tumor progression, the pro-tumor and anti-tumor effects of NETs and the involvement of DNases in the regulation of DNA-mediated metastasis and tumor dissemination.

## 2. Brief Historical Insight

Horizontal DNA transfer is not particularly widespread; there is some experimental evidence showing that this phenomenon is not frequent and is difficult to prove. Horizontal DNA transfer was first shown in bacteria, in which this process plays an important role in the development of antibiotic resistance and adaptation to new environments [19,20]. The exchange of genetic material between cells in plant tissue grafts is also known [21]. Many studies published between 1960 and 1970 indicate that eukaryotic cells can absorb extracellular DNA [22,23], and the ability to exchange DNA does not depend on the origin of the donor and recipient cells [22]. Nevertheless, it has been shown that exogenous DNA is capable of inducing chromosome damage [24] and changing the karyotype of cells in the case of a different origin of exogenous DNA and the recipient cells [12]. There is also evidence that DNA captured by mammalian cells undergoes replication, transcription and translation [25].

Stroun, Anker and Gahan developed the concept of the exchange of genetic material: in experiments with bacteria, plants and higher organisms, they identified complexes containing bacterial DNA and their DNA- and RNA-dependent DNA polymerases, capable of integrating fragments of the bacterial genome into the genome of the host cell [26,27]. Based on this finding, these authors proposed the concept of virtosomes–lipoprotein complexes containing DNA, RNA and DNA- and RNA-dependent DNA polymerases that play an important role in intercellular communication [28]. Considering the existence of horizontal DNA transfer, the presence of large amounts of tumor-derived cfDNA in the blood of patients with cancer suggests their possible function as carriers of certain ‘tumorigenic’ properties to normal cells.

## 3. Composition of cfDNA

Leon and colleagues [29] first reported that cfDNAs are present in the blood serum of patients with cancer in concentrations exceeding those in the blood of healthy donors; subsequent studies have supported this finding [30,31,32,33]. The concentration of cfDNA in the blood of patients with cancer can increase to 1000 ng/mL and more, and on average it ranges from 200 to 300 ng/mL [34,35,36]. It has been firmly established that cfDNA contains fragments of oncogenes, hypermethylated tumor suppressor genes, aberrant microsatellites, aberrant DNA methylation genes and rearranged chromosomes [37,38]. Most of this cfDNA has a tumor origin, and it has been suggested that such cfDNA appears in the blood as a result of necrosis and apoptosis of tumor cells, cells of the tumor microenvironment destroyed during the processes of tumor growth, or immune cells, mobilized to eliminate tumor cells. Researchers suggest that cfDNA derived from tumor cells must be perceived not just as ‘half-eaten’ fragments of dead cells, but as agents with a certain pathogenic function.

It has been established that circulating cfDNA contains sequences not typical for normal cells, including hypermethylated sequences [39], mutant mitochondrial DNA sequences [40,41], retrotransposons [42,43,44] and even rearranged genes [45]. Mutant sequences of oncogenes and oncosuppressors have frequently been found. Fragments of the mutant *KRAS* gene, which is a key participant in the intracellular cascades responsible for cell oncotransformation, and the *TP53* gene, which plays a key role in triggering apoptosis, are the most frequent in the pool of cfDNA in patients with cancer. Mutant sequences of the *EGFR*, *BRAF*, *HER2*, *ALK* and *BRCA* genes, among others, are often found in the blood of patients with various types of cancer [46]. For some genes, an increase in the number of copies has been found both in tumor cells and in the cfDNA pool. In patients with intracranial tumors, an increased representation of the *HER2* gene fragments was found in the cerebrospinal fluid [47]. High levels of fragments of the *MYC* gene were found in the blood of patients with neuroblastoma [48,49].

In the cfDNA pool of patients with cancer, many authors have found an increased number of MGE including short interspersed nuclear elements (SINEs), mostly Alu, and long interspersed nuclear elements (LINEs), in particular, L1 and L2 [50,51,52]. MGE are repeating elements of the genome that can be located in tandem (satellite heterochromatin, telomeres, etc.) and can be scattered throughout the genome (MGE, pseudogenes, etc.) [53]. In 1977, researchers first reported the enrichment of cfDNA with Alu fragments in patients with cancer, at a significantly higher level than in healthy donors [2], and this has been confirmed many times [54,55]. The increased level of Alu repeats in circulating cfDNA is often correlated with the stage of tumor progression and with its severity [56]. The amplification and insertion of LINEs, some of which are actively translocating elements, have been noted in lung cancer, colon cancer and brain tumors [57]. The presence of these sequences in the blood of patients with cancer indicates both the processes occurring in the tumors and the fact that these DNA could be involved in signal transmission through MGE.

Naked cfDNA is not found in the blood; short DNA fragments circulate in complexes with histones and are often found in nucleosomes as well as in aggregates with blood proteins [58]. Large DNA is often found in more multiplex protein complexes and membrane particles, such as exosomes, apoptosomes, virtosomes and other MVs [59]. An increase in the amount of cfDNA in the complexes indicates a disruption in the normal functioning of proteases and nucleases; the presence in the blood of an excessive amount of DNA-containing MVs, predominantly of tumor origin, indicates the existence of signal transmission from tumor cells to normal cells in the form of genes or their promoter regions.

## 4. Participation of cfDNA of Tumor Origin in Malignant Transformation of Healthy Cells

The ‘classic’ theory of metastasis assumes that metastases are formed by the cells of the primary tumor spreading through the bloodstream, with the formation of metastatic foci in the most ‘sensitive’ target organs. In 2010, a group of researchers led by Garcia-Olmo put forward a hypothesis on the key role of cfDNA in metastasis (‘the hypothesis of genometastasis’). It is assumed that metastases are formed as a result of transfection of normal cells located in the target organs with tumor-derived cfDNA [10]. This hypothesis has been adopted by other authors as a model to explain the contradictions in the experimental data relating to metastasis.

To prove their hypothesis, Garcia-Olmo and colleagues used the blood plasma of patients with colon cancer as the source of cfDNAs containing mutant fragments of *KRAS*, *TP53*, and *HBB* (encoding β-globin) genes. NIH-3T3 murine tumor cells lacking such a mutant gene pattern were incubated for 20 days with blood plasma from patients with colon cancer. They were then injected subcutaneously into NOD-SCID mice and demonstrated high oncogenic potential. In tumor tissue developed from ‘transformed’ NIH-3T3 cells, mutant sequences of the *KRAS* gene were found. Hence, the authors concluded that the malignant transformation of murine cells was achieved by the transfection of cfDNA from a human blood plasma. In fact, they proved the acquisition of the malignant tumor phenotype by the murine cells. However, in a similar experiment with human adipose tissue stem cells (hASC) as recipients of cfDNA from the same blood plasma of patients with cancer, no mutant forms of any of the studied genes in the cells were found, and the formation of tumors by these cells in mice was not detected [11].

In 2012, Trejo-Becerril and colleagues showed that cfDNA can induce colon tumors in Hsd:Wistar rats and BALB/c mice. A tumor-derived fragment of the *KRAS* gene with point mutations in the twelfth codon not only causes the transformation of recipient cells in vitro, but also promotes tumor growth in vivo [12]. Similar results have been obtained by other groups using apoptosis-destroyed cells as a source of exogenous DNA. Bergsmedh and colleagues proved the transfer of human *HRAS* and rat *Cmyc* gene fragments to recipient mouse embryonic fibroblasts [13]. Gaiffe and colleagues also demonstrated malignant transformation using HIV-positive cervical cancer cells and human mesenchymal cells as the donor and recipient, respectively [14].

In addition to oncogene fragments, MGE delivery into recipient cells could cause malignant transformation. Substantial evidence has been accumulated about malignant transformation of cells by retrotransposition. Mutations of protooncogenes and oncosuppressors caused by the insertion of retrotransposons can induce tumor development [60,61]. The insertion of full-length SINE-VNTR-Alu retrotransposons (SVT) into intron 8 of the caspase-8 gene (*CASP8*) is associated with an increased risk of basal cell carcinoma and breast cancer but reduces the risk of prostate cancer [62]. To date, there is almost no reliable data that the insertion of SINEs, LINEs or other MGE is associated with metastasis, but already accumulated data indicate that such a connection exists. Thus, an increase in the level of SINEs and LINEs in blood cfDNA during the development of melanoma B16, Lewis lung carcinoma (LLC) and drug-resistant lymphosarcoma RLS_40_, correlated with the number of metastases. A decrease in the number of metastases was observed in response to DNase I-based therapy and was accompanied by a proportional decrease in the levels of SINEs and LINEs in the blood of tumor-bearing mice [63].

## 5. Routes of Tumor-Derived cfDNA Penetration into Cells

The question of how DNA penetrates vulnerable cells remains unclear. Mammals have the Toll-like receptor 9 (TLR9) that is capable of recognizing DNA [64]. These receptors are expressed by immune-competent cells like monocytes, macrophages, plasmacytoid dendritic cells and B lymphocytes [65]. However, TLR9 is an intracellular protein, localized in endosomes, with a role to recognize unmethylated CpG of bacterial, viral and mitochondrial DNA [66]. On the cytoplasmic membrane of the tumor cell itself, there are CCDC25 receptors capable of recognizing DNA within NETs [67]. In vitro DNA binding assays have illustrated that CCDC25 preferentially binds NET-DNA with a high level of 8-hydroxy-2′-deoxyguanosine (8-OHdG, a hallmark of NET-DNA) [68]. However, at the moment, no receptors have been found on the surface of normal cells capable of recognizing DNA, although most cells are able to bind DNA due to electrostatic interactions [69].

Because cfDNA often circulates in complexes with proteins and lipoproteins, these structures can also interact with various receptors and facilitate the penetration of cfDNA into cells [70]. Thus, it has been shown that nucleosomes can bind to cell surface proteins with molecular weights of 29 and 69 kDa [71,72,73]. In addition, it seems that antibodies or complement system factors like C1q bound directly to DNA or to histones can interact with cell surface proteins [74,75]. DNA endocytosis occurs actively in immune cells, but it is unclear whether other cells are capable of capturing DNA [76].

The discovery of exosomes and other membrane MVs pushed the development of the theory of horizontal cfDNA transfer. Double-stranded DNA (dsDNA) in MVs was first detected in 2013; later, it was shown that MVs may contain DNA fragments longer than 10 kb [77,78]. Moreover, recent research has also shown that more than 93% of plasma cfDNA is present in exosomes [79]. In addition to being encapsulated in exosomes, cfDNA also can be secreted out of cell through an amphisome-dependent mechanism [80]. There is a large body of data about MVs containing mutant or overamplified DNA fragments in the blood of patients with various forms of cancer; the fragments are typical for the specific types of cancer and often occur in metastases. Circulating MVs from the blood of patients with prostate cancer are characterized by enrichment with fragments of mutant *MLH1*, *PTEN* and *TP53*, whereas circulating MVs of patients with pancreatic cancer are enriched with mutant *KRAS* and *TP53* [81]. Considering that MVs are important messengers between normal cells, the presence of cfDNA in MVs of tumor origin may be a key factor in the transmission of the metastatic signal.

The incubation of epithelial cells with MVs containing fragments of the *HRAS* gene led to the transfer of the full-length gene into recipient cells, which stimulated phenotypic changes and active cell proliferation [82]. In 2013, Cai and colleagues succeeded in transferring hybrid DNA containing *BCR*/*ABL* sequences encapsulated in chronic myelogenous leukaemia (CML) cell-derived MVs to human embryonic kidney (HEK293) cells and neutrophils [77]. A similar result was obtained by this group with vascular wall smooth muscle cell (VSMC)-derived MVs containing the angiotensin receptor type 1 (*AT1R*) gene, which successfully delivered *ATR1* to HEK293 cells, as well as leucocyte-derived MVs with the *SRY* gene, which delivered *SRY* DNA into endothelial cells [82,83].

## 6. Pro-Tumor and Antitumor Effects of NETs

NETs are formed and released in the extracellular space during a process called NETosis, which is used by the innate immune system to destroy and eliminate pathogens that have entered the body [64,84,85]. During the controlled release of DNA, network-like structures are formed; they are decorated with various components of neutrophil granules, such as neutrophil elastase (NE), myeloperoxidase (MPO) and other reactive oxygen species (ROS) producers, interleukins (IL) and other proteins [86]. NETs are the important component of the antimicrobial action of neutrophils, namely the ability to capture and kill pathogenic organisms and to induce inflammatory changes [87].

Over the past decade, a lot of data have been collected on the relationships between NETs and tumors, and these data are rather controversial [88]. The earliest data indicated the presence of an excess amount of newly formed NETs in the tumor microenvironment, both in tumor-bearing mice with various types of tumors and in patients [89]. To date, there is evidence that NETs, on the one hand, can promote both tumor growth and metastasis development, while on the other hand, they contribute to tumor clearance [90]. However, for a long time it remained unclear whether the development of the tumor causes the recruitment of an excessive number of neutrophils, or whether the neutrophils and NETs themselves somehow contribute to the development of the tumor and metastasis.

An excessive number of neutrophils and increased NET formation have been found in many experimental tumors in mice, as well as in biopsies of tumor tissue from patients [91]. Large accumulations of dead neutrophils and NETs have been found in the microenvironment of haemorrhagic lungs in LLC mice, Ewing’s sarcoma, small bowel cancer, colorectal cancer, breast cancer and lymphoma [92,93,94,95,96,97]. Patients with advanced lung, breast or gastric cancer are characterized by excessive formation of NETs and, moreover, research also indicates that NET levels may be associated with metastasis [98,99].

Some authors suggest that NET deposition in tumor tissue may have a cytotoxic effect. NETs were found to inhibit cancer cell growth by inducing apoptosis of Caco-2 and AML cells [100], as well as the migration and viability of melanoma cells [101]. In a CT-26 mouse intestinal adenocarcinoma model, the oncolytic vesicular stomatitis virus triggered an inflammatory response that included neutrophil-dependent initiation of coagulation in tumor blood vessels, possibly mediated by NETs [102].

The overwhelming majority of studies, however, have confirmed that NETs promote tumor progression. Correlations between the stage of oncological disease development and NET formation have continually been shown. A study comparing triple-negative human breast cancer (oestrogen− progesterone−, HER-2−) with luminal HER-2+ breast cancer found excessive NET formation in the case of triple-negative human breast cancer that correlated with the spread of metastases [103]. In patients with stage III/IV gastric cancer, the NET density was significantly higher compared with healthy donors and patients with stage I/II of the disease, while no significant differences in NET density were found between patients with stage III or IV of the disease [98]. The late stages of breast and lung cancer were characterized by higher NET density compared with the early stage [99,103]. Moreover, NETs can serve as an independent predictor of pancreatic duct adenocarcinoma (PDAC): high NET density corresponds to poor postoperative survival in patients with PDAC [104].

**Figure 1:**
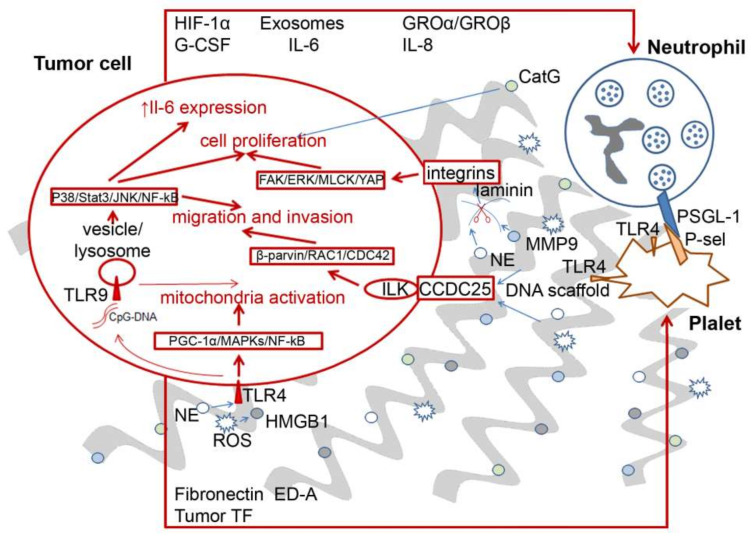
Interactions of neutrophils, platelets and tumor cells in tumor development. Tumor cells can directly induce NETosis (the formation of neutrophil extracellular traps (NETs)) by secreting factors such as exosomes, growth-regulated oncogenes (GRO), granulocyte-macrophage colony-stimulating factor (GM-CSF), hypoxia-inducible factor 1α (HIF-1α) or others. Neutrophil activation causes NETosis and NET release. Different NET components are capable of inducing tumor growth and migration. DNA serves as a scaffold and trapping component, additionally acting through CCDC25 receptor binding. HMGB1, a DNA-binding protein, and components of neutrophil granules, namely neutrophil elastase (NE) and reactive oxygen species (ROS), activate tumor cells via the TLR4–TLR9 pathway. NE and matrix metalloproteinase 9 (MMP-9) cut laminin, which through integrin binding induces cascades that result in tumor cell proliferation. Other neutrophil components such as cathepsin G (CatG) or proteinase 3 are also apparently capable of activating tumor cells. In addition, neutrophils are capable of activating platelets through P-selectin glycoprotein ligand-1 (PSGL-1)–P-selectin (P-sel) interactions with TLR4, leading to cancer-associated thrombosis. PSGL-1–P-sel interactions also cause NET release. Furthermore, the tumor promotes platelet activation through the production of tissue factors (TF) such as fibronectin ED-A; TLR4 pathways also mediate these actions.

More details showing interactions and crosstalk between tumor cells, neutrophils and NETs are depicted in Figure 1. Many experimental data obtained indicated that cancer cells can stimulate NET production, and vice versa. *In vitro*, breast cancer, diffuse large B-cell lymphoma and non-small cell lung cancer cells can stimulate neutrophils to release NETs by secreting cytokines such as IL-6, IL-8, granulocyte-macrophage colony-stimulating factor (GM-CSF), exosomes and hypoxia-inducible factor 1α (HIF-1α) (Figure 1) [88]. Further tumor growth leads to damage of surrounding tissues and intravascular thrombosis of the tumor, and, consequently, ischaemic necrosis. Together, these factors can drive the formation of NETs [88].

In turn, NETosis can provoke tumor growth and metastasis. In a model of small intestine cancer, it was found that increased concentrations of circulating lipopolysaccharides (LPS) increase the expression of the complement component C3a, which leads to NETosis, the induction of coagulation and, in turn, stimulates oncogenesis [105]. Coagulation-induced clots create an attractive environment by inducing a positive feedback loop and by promoting the production of neutrophils, which stimulate coagulation, which then further stimulates tumor development [105]. Some factors secreted by neutrophils, such as IL-17, can promote the epithelial–mesenchymal transition (EMT) of tumor cells, which promotes the invasion of tumor cells into the blood or lymphatic vessels and the subsequent formation of distant metastases [98]. Various NET components including matrix metalloproteinase 9 (MMP-9), cathepsin G and NE can also stimulate the growth and adhesion of tumor cells (Figure 1). As a result, NETs may allow for the formation of a specific tumor microenvironment that promotes the growth of tumor cells. These events can promote proliferation, inhibit apoptosis and induce metastasis [88].

It has recently been demonstrated that NETs can promote metastasis by sequestering tumor cells in mice with human A549 lung carcinoma and mouse LLC [106]. In a model of systemic infection (conditions suitable for metastasis), the release of NETs into the microvasculature and the subsequent uptake of circulating cancer cells by DNA networks has been shown. The authors suggested that, similarly to the capture of bacteria, tumor cells can be immobilised by NETs. Indeed, tumor cells captured by NETs have been shown to survive and proliferate, forming pseudometastatic clots. NETs or their degradation products probably inhibit immune cells, ensuring the survival of the captured tumor cells. The increase in NET density increases metastasis development, and the presence of intravascular NETs after sepsis promotes metastasis in mice [106].

In addition to the direct effect of NETs on the tumor, NETs also contribute to the occurrence of systemic pathological effects in oncological diseases. Indeed, NETs have been hypothesized to contribute to the development of deep vein thrombosis [96]. Cancer-associated thrombosis is one of the most common causes of death in patients with cancer. In 2012, it was shown, for the first time, in models of chronic myelogenous leukaemia and carcinoma of the mouse mammary gland that late stages of the disease are accompanied by excessive NETosis occurring simultaneously with the development of venous thrombi in the lungs [96]. As mentioned earlier, NETs promote platelet aggregation mainly due to negatively charged DNA, as well as histones that stimulate thrombin formation [107]. Moreover, both tissue factors and factor XII are inducers of external and internal coagulation pathways and are often present in NETs [108,109,110].

Excessive NETosis can also lead to complications not directly related to tumor growth, but often occurring in patients with cancer. Recently, in models of breast carcinoma and murine insulinoma, it has been demonstrated that NETs promote vascular dysfunction and systemic inflammation in organs other than tumor growth sites and areas of metastasis, such as the heart and kidneys [111]. Renal vascular hypoperfusion and associated inflammation are indicators of renal failure, a common problem in patients with cancer, often with fatal outcomes [112,113,114]. In 2016, it was shown that NETosis and the vascular dysfunction caused by it are often found in patients with ischaemic stroke complicated by overt or latent oncological disease [115].

To summarize, NET release in early stages of oncological disease may promote tumor elimination, but in the late stages the destruction of excessive NETs is critical for saving the patient’s life. Therefore, the development of drugs aimed at limiting the recruitment of neutrophils, blocking NETosis and the destruction of newly formed NETs is an urgent task.

## 7. DNases and Their Role in the Maintenance of cfDNA Homeostasis under Pathological Conditions

Due to the potential dangers posed by persistent cfDNA and NETs to the body, the removal of cfDNA from circulation is vital for homeostasis. The half-life of cfDNA is quite short, estimated to be between 16 min and 2 h [116]. There are at least three potential mechanisms of DNA excretion from the blood: active uptake by the reticuloendothelial system in the liver and spleen, passive filtration by the renal system and direct degradation by nucleases [117].

### 7.1. Intracellular DNases

The level of cfDNA is affected markedly by intracellular nucleases involved in DNA decay during apoptosis, necrosis and other types of cell death. The most important are DNase X and DNA fragmentation factor B (DFFB, DFF40 or CAD) (Table 1). Biochemical suicide molecule endonuclease DNase X was the first DNase I-like enzyme to be discovered. DNase X belongs to the DNase I-protein family, which comprises DNase I, DNase X (DNase I-like 1), DNase I-like 2 and DNase I-like 3 (DNase IL3). The DNase X gene is located on q28 of the X chromosome, and the protein itself is highly conserved, which suggests its key importance for the maintenance of cellular integrity [118]. DNase X is expressed actively in almost all cells to suppress alien gene transfer [119]. DNase X is Ca^2+^-, Mg^2+^-dependent and cleaves chromatin with the formation of oligonucleosomes with double-strand breaks (Table 1). DNase X contains a conserved C-terminal hydrophobic domain, due to which DNase X is able to anchor in the cell membrane or in the nuclear envelope [119,120]. The presence of DNase X on the outer membrane has an immunological function, protecting the cell from alien bacterial, viral and other DNAs [121].

In some cases, an increase in the concentration of DNase X can occur in cancer cells, but the reasons for this are not fully understood [122]. Abnormal overexpression of DNase X has been detected in cervical intraepithelial neoplasia (CIN), cervical carcinoma and oral squamous cell carcinoma (OSCC) [123,124]. In the cytoplasm of tumor cells, DNase X is in an inactive form due to the accumulation of a specific antibody to DNase X, an epitope of the Apo10 protein, and does not degrade the DNA of tumor cells [124]. According to other studies, most DNase X just moves to the cell surface, and the protection of tumor cells from alien DNA does not occur due to its degradation, but due to cell shielding [125]. Thus, cell shielding and the accumulation of antibodies to DNase X block apoptosis, and this is a common process for the development of pre-malignant and malignant tumor cells [126].

DFFB is a caspase-3-activated apoptotic nuclease triggering chromatin fragmentation during apoptosis. DFFB cleavage results in formation of oligonucleosomes with double-strand breaks (Table 1). DFFB is expressed by almost all cells and usually is bound with its inhibitor DFFA and activated by the cleavage of its inhibitor by caspase-3 [127]. DFFB is Mg^2+^ dependent and cleaves internucleosomally both chromatin and naked DNA, preferably at 5′A(G)→3′X >> 5′C(T)→3′X, where X is any nucleotide [127]. The presence of a large number of such fragments in the cfDNA pool indicates that the sources of such cfDNA are apoptotic cells.

Mutations in the *DFFB* gene lead to the acute disruption of normal cell cascades, the disruption of DNA repair and mutagenesis similar to malignant transformation [131]. Only one high-frequency mutation site in the *DFFB* gene has been found in patients with endometrial cancer; this mutation led to a violation in the expression of a large number of other genes [132].

Endonuclease G (EndoG), a pro-apoptotic mitochondrial endonuclease, also impacts DNA homeostasis. EndoG is Mg^2+^/Mn^2+^-dependent and cleaves single-stranded DNA (ssDNA) and dsDNA in chromatin and DNA in DNA/RNA heteroduplexes with a preference for G- and C-rich regions with formation of oligonucleosomes with double-strand breaks and internal single-strand nicks (Table 1) [129]. Zhdanov and colleagues showed that the overexpression of EndoG can increase the expression of other DNases, including DNase I, DNase X and DNase IL3 [133]. This overexpression was mostly associated with DNA degradation in the promoter/exon 1 regions of the endonuclease genes [133], although EndoG is also able to participate in alternative splicing of DNase I mRNA [134]. Interestingly, the overexpression of EndoG is important for the development of tumor progression, and the silencing of the EndoG gene contributes to the suppression of the proliferation of tumor cells [135,136].

**Figure 2:**
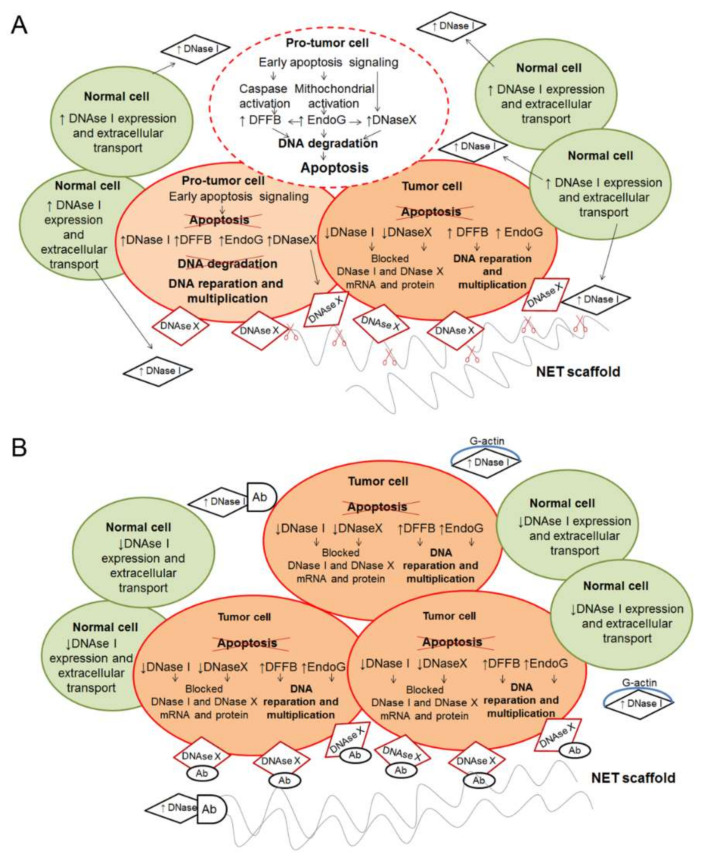
Changes in the activity of DNases in the early (**A**) and late (**B**) stages of tumorigenesis. (**A**) A cell with a pro-tumor phenotype accumulates changes that trigger apoptosis, including activation of EndoG, DFFB and DNase X synthesis. EndoG activates other DNases. In the case of a successful start of the apoptotic cascade, intracellular DNases and later DNase I participate in apoptosis by destroying damaged DNA. At the same time, cells of the tumor environment produce and secrete DNase I into the intercellular space, which should also promote apoptosis. If apoptosis fails, pro-tumor cells accumulate DNases, which are either present in an inactive state in the cytoplasm or are accumulated on the surface of the cell (predominantly DNase X). Surface nucleases protect tumor cells from exogenous DNA and DNA from NETs, thereby preventing the catch and destruction of tumor cells during the early stages of tumorigenesis. EndoG and DFFB take part in the processes of DNA repair and amplification, and mostly remain highly active. The total effect leads to an increase in DNase activity in tumor cells and in the tumor microenvironment. (**B**) During cell transformation, the EndoG and DFFB levels in tumor cells remain high and promote tumor growth. Tumor cells accumulate inactivated copies of DNase I and DNase X on its surface and in intercellular space. DNase I is inactivated by binding with actin filaments (mostly G-actin) and anti-DNase antibodies. Neighboring cells also reduce DNase I production. Overall, these changes lead to a decrease in DNase activity in the tumor microenvironment that reduces the apoptotic pressure on tumor cells. In addition, a low level of DNase activity leads to the accumulation of NETs; the development of inflammation; an increase in the functioning of transformation cascades in the tumor cell; as well as their detachment, migration and metastasis.

The possible role of DNases in tumor development is shown in Figure 2. In the early stages of cell transformation, EndoG, DNase X and DFFB are activated when apoptosis is triggered. Neighbouring normal cells contribute to this process by activating the expression of the genes of secreted DNases (DNase I and DNase IL3). The increase in the concentration of secreted DNases in the surrounding space attracts macrophages, which impact the antitumor immune response and lead to increased apoptosis of protumor cells (Figure 2A). In the case of impaired apoptosis, EndoG participates in other cascades, changing the cell phenotype and leading to malignant transformation; moreover, DFFB stops synthesizing or becomes inactive. Excessive DNase X is accumulated on the cell surface and secreted into the intracellular space, where it performs a protective function, and some of the inactivated DNase X molecules are accumulated inside the tumor cell (Figure 2A). DNase X on the surface of the tumor cell protects it from destruction by NETs and can also contribute to safe trapping and further formation of metastases. However, at the later stage of cell transformation, the DNase activity decreases sharply (Figure 2B). EndoG continues to function in an excess mode, but it is involved primarily in DNA repair, protecting the cell from apoptosis. Thus, it is obvious that DNases are crucial for the maintenance of normal homeostasis of cells and the whole organism.

### 7.2. Extracellular Secreted DNases

Extracellular secreted DNases (exDNases) are the main tools for the removal of excess circulating cfDNA. For the most part, exDNases are secreted by endothelial cells, and in some cases by bacterial cells of the intestine microbiome [137]. In blood there are generous amounts of enzymes capable of destroying cfDNA including DNase I and DNase IL3, and also more rare nucleases such as EndoG, apoptosis-inducing factor (AIF), topoisomerase II and cyclophilins [138]. Two circulating DNases, DNase I and DNase IL3, have been shown to have a significant effect on cfDNA levels and/or the enrichment of certain cfDNA fragments [117]. The main role of circulating DNases in an organism is the digestion of the DNA received with food in the intestinal tract. Beyond the digestive role, DNases protect the intercellular space against autoimmune pathological action of cfDNA [139]. Decoding the role of nucleases in the regulation of the level/enrichment of cfDNA is difficult due to the presence of various nucleases in the human body and the ability of nucleases to act both in generating cfDNA through cell death pathways such as apoptosis and in the clearance of cfDNA at the blood level. A deficiency of a particular nuclease can be masked by the action of other nucleases [117].

The main DNA-hydrolyzing enzyme in blood is DNase I belonging to the DNase I protein family. DNase I is expressed predominantly in exocrine cells in the gastrointestinal tract, salivary glands, kidneys and vascular endothelial cells. DNase I is Ca^2+^/Mg^2+^-dependent and Zn^2+^-sensitive. It preferably cleaves naked dsDNA but can cleave ssDNA and DNA in DNA/RNA heteroduplexes less efficiently. It is able to hydrolyze chromatin preferentially with the specificity 5′-T > C >> A,G→3′X, where X is any nucleotide (Table 1) [140]. Normally, the enzyme activity is only 4.4 ± 1.8 units/L (expected activity 6–20 units/L) of blood serum, which is explained by the presence in the blood of a natural inhibitor of DNase I, namely actin (up to 100 μg/mL) [141]. DNase I cleaves DNA to small fragments such as tetranucleotides; it is a single DNase that cleaves DNA with the formation of mononucleosomes [140].

Another DNase mostly recruited to destroy cfDNA is DNase IL3, also belonging to the DNase I family [142]. DNase IL3 is mainly expressed in the liver and spleen, vascular endothelial cells, macrophages and dendritic cells (Table 1). Unlike DNase I, DNase IL3 has a positively charged amino acid sequence in its C-terminal region that allows its transfer to the nucleus, provides high affinity to lipid membranes and facilitates its encapsulation in MVs [143]. It is postulated that this sequence provides the ability of DNase IL3 to digest protein-associated DNA in addition to naked DNA [144]. Therefore, DNase IL3 can degrade chromatin-associated DNA more efficiently than DNase I, which preferably ‘eats’ naked DNA [145]; DNase I is more efficient in cleaving naked DNA [144]. Apparently, DNase IL3 is not inactivated by actin in the cytoplasm or in the blood [117]. The data obtained indicate that DNase IL3 should be recruited actively during apoptotic and inflammatory processes at relatively early stages, before DNase I. The presence of large fragments (>1000 bp) in the case of necrotic processes indicates a violation of the normal expression of DNase IL3.

For the normal removal of cfDNA from the intercellular space and blood, the action of both DNases is required. DNase I exhibits high efficiency in the cleavage of naked DNA and only slightly cleaves DNA in nucleoproteins, whereas DNase IL3 demonstrates less efficiency in cleaving naked DNA, but high efficiency in cleaving DNA in nucleoprotein complexes. It can be suggested that there are some compensatory mechanisms that make it possible to change the activity of nucleases in the case of failure of any of them or their knockdown. Likewise, both nucleases may be involved in NET clearance: the shutdown of both nucleases, but not each separately, leads to uncontrolled NETosis during sepsis or myeloproliferation [146].

### 7.3. DNase Activity of the Blood during Tumor Progression

Many pathological conditions associated with the release of cfDNA are characterized by changes in the DNase activity of the blood, which indicates the change in the expression of secreted nucleases. The total DNase activity of the blood has been found to differ between healthy donors and patients with cancer, and often depends on the type of cancer and its stage. The earliest data showed that patients with malignant lymphomas were characterized by decreased DNase activity, whereas patients with breast cancer demonstrated higher levels of DNase activity in comparison with healthy donors [147]. Patients with oesophageal cancer or prostate cancer are also characterized by extremely low blood DNase activity, while the level of cfDNA is significantly increased [148,149]. For patients with liver cancer, a correlation has been found between an increased level of DNase activity in the blood with a low concentration of cfDNA in the preclinical setting and the risk of developing cancer. Nevertheless, tumor progression in patients resulted in a decrease in the activity of blood DNases, which is accompanied by an increase in the level of cfDNA [150].

Apparently, an increase in the cfDNA concentration provokes an increase in blood DNase activity. Thus, an increase in DNase activity was observed in precancerous conditions and at an early stage of the development of oncological diseases [151]. However, with the development of the disease, DNases are inactivated when they bind to G-actin and anti-DNase antibodies, therefore, decreasing DNase activity [152]. These data indicate ambiguous relationships between tumor-derived DNA/secreted DNases and tumor progression/intracellular DNases.

## 8. Exogenous DNases as Antimetastatic and Antitumor Agents

Studies of DNases as antimetastatic and antitumor agents have been conducted for a long time; however, despite the obvious success, the collected data obtained are few and fragmented. Bovine pancreatic DNase I (bpDNase I) has been investigated widely to treat viral and autoimmune diseases, including those caused by the herpes virus, adenoviruses and other DNA viruses [153]. The antitumor activity of bpDNase I was first demonstrated in a mouse Ehrlich ascites carcinoma model in 1961 [154]. In 1964, the first information on the antitumor activity of bacterial DNase from *Serratia marcescens* was available [155]. The antimetastatic potential of bpDNase I was shown in the 1960s: researchers used a model of spontaneous lymphocytic leukaemia of AKP mice and showed that 250 mg/kg body weight of DNase I decreased the size of the degenerated lymph nodes of affected animals [156]. Twenty years later, a pronounced antimetastatic effect of bpDNase I was shown in the L5178Y-ML mouse tumor model metastasizing in the liver [157]. In 2009, the Alvarado-Vásquez group demonstrated the ability of this DNase to inhibit the proliferation of several tumor cell lines (Calu-1, SK-MES-1, HeLa, HEp-2 and L-929), while normal peripheral blood mononuclear cells (PBMCs) and human embryonic fibroblasts were not affected [158]. In addition to inhibition of tumor cell proliferation, bpDNase I caused a significant decrease in cfDNA in the culture medium [158]; this finding indicates the importance of its DNase activity and the fact that it targets several types of DNA.

Despite the revealed antitumor and antimetastatic activity of DNase I, the mechanism of this activity remains unclear. The accumulated data about the ability of tumor-derived cfDNA to provoke metastases and participate in NET formation suggest that the antitumor effect of DNases is associated with their main function: the ability to cleave DNA, including DNA involved in tumor development. In the last decade, there has been a renaissance in the study of the antitumor potential of DNases. In 2010, the antimetastatic potential of bpDNase I was demonstrated in two mouse models, LLC and A1 hepatoma, when DNase was administered intramuscularly daily at doses from 0.02–2.3 mg/kg body weight [159]. A correlation was found between the antimetastatic effect of DNase I and the decrease in the level of cfDNA in the blood plasma of tumor-bearing animals [159]. In 2013, Wen and colleagues showed high antimetastatic potential of bpDNase I in an in vivo model of pancreatic cancer [160]. In an in vitro study, bpDNase I reduced the migration of pancreatic cancer cells and destroyed tumor-specific NETs, changes that would lead to a decrease in the migration of tumor cells in vitro and the level of metastasis in vivo.

Further research has been divided into two main directions: the destruction of circulating tumor-derived cfDNA, which may contribute to metastasis, and cfDNA integrated into the NET facilitating migration and invasion. It has been shown that bpDNase I destroys excess NETs, reducing their contribution to pathogenic processes like airway inflammation and pneumococcal meningitis [161,162]. In vitro, during the cultivation of neutrophils with lung carcinoma cells in the presence of bpDNase I, the adhesion ability of the cells was increased significantly, while without DNase, but in the presence of neutrophils and, therefore, NETs, the migration and adhesion of tumor cells were five times higher compared with intact cells [97]. In gastric cancer cells cultured in vitro, the EMT-promoting effect of NETs was eliminated by a DNase-1/PAD4 inhibitor [98]. It should be noted that NETs are multicomponent targets. Therapeutic agents targeting the destruction of NETs can be found in the review of Urszula Demkow [163].

In vivo, in the LLC model, bpDNase I blocked the ability of NETs to capture circulating tumor cells (CTCs), followed by an increase in their migration to the liver [164]. Intraperitoneal injections of bpDNase I in mice with a human pancreatic tumor resulted not only in a decrease in NETs, but also in a decrease in the intensity of pathological thrombosis [165]. Injections of microparticles with immobilized bpDNase I also led to a reduction in the total NET area in mice with human lung cancer, which correlated with a decrease in the level of metastasis [103]. In the LLC model, bpDNase I together with decreased metastasis, increased DNase activity and destroyed cfDNA in the bloodstream of tumor-bearing mice [166], and its main targets in cfDNA are tumor-associated DNA, such as tandem repeats, fragments of MGE (SINEs and LINEs) as well as fragments of oncogenes [166]. The antimetastatic and antitumor effect of bpDNase I and its ability to destroy tumor-derived cfDNA has been demonstrated in murine models of melanoma B16 and resistant lymphosarcoma RLS_40_, which indicates the general patterns of the DNase I functioning in metastases and tumor inhibition [63]. Thus, DNase I has attracted the attention of researchers not only as a ‘restorer of the balance’ between the concentrations of cfDNA and the DNase activity of blood plasma, but as a specific agent for the destruction of tumor-derived cfDNA that could contribute to metastasis formation.

**Figure 3:**
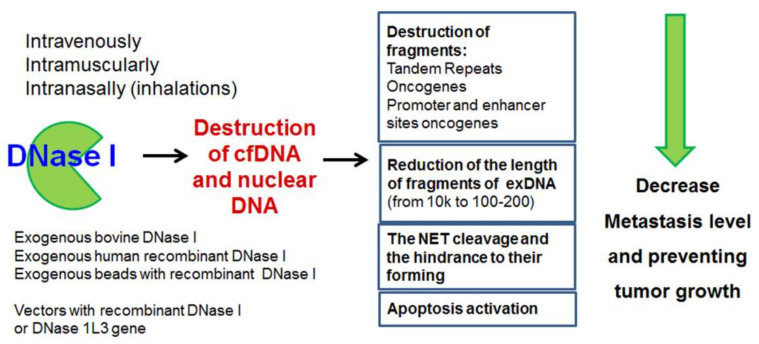
Possible use of DNases as therapeutic agents. Abbreviation: NET, neutrophil extracellular trap.

The inhibition of tumor growth by exogenous DNases is a good strategy to replace secreted DNases and countervail its function in the maintenance of normal DNA homeostasis of the organism (Figure 3). One of the prospective directions is the restoration of the normal level of DNase expression in cells. Karli Rosner suggests applying DNase I as an individual anti-cancer therapeutic. The scheme of the suggested therapy is: (i) the expression of a vector encoding genetically modified DNase I that escapes inactivation by G-actin; (ii) an increase in the amount of DNase I in tumor cells; (iii) the introduction of double-stranded breaks in intracellular DNA, and the triggering of apoptosis. There was a significant reduction in the number of cells of a chemo- and radioresistant line of human melanoma under the action of DNase I [167]. In many types of tumors, a high density of DNase X/Apo10 antibodies has been found, which indicates the inactivation of DNase X in tumors [124]. Based on this, the restoration of the level of DNase X in tumor cells might be a useful goal for individually targeted therapy. A strategy for the delivery of transgenic vectors encoding several DNases such as DNase I, DNase IL3, DNase II and DFFB under a common promoter was very successful in triggering apoptosis in cancer cells [168]. Delivery of the DNase I gene in an adenoviral vector to liver cells led to the suppression of liver metastases by destroying local excessive NET production [169].

The combined action of DNase I with other compounds is also of interest. DNase I in combination with hydroxychloroquine (HCQ) or aspirin was shown to be more effective than when used alone in inhibiting NET formation and decreasing hepatocellular carcinoma metastasis [170]. The administration of DNase I and proteases (papain, trypsin and chymotrypsin) reduced both metastases and the primary tumor node [103]. The use of DNase I together with NET inhibitors (PAD4, NE) was effective for cancer therapy [171].

Commercially available recombinant human DNase I (dornase alfa, trade name Pulmozyme) was developed the treatment of cystic fibrosis and acute bronchial asthma, as an agent that reduces the viscosity of sputum in patients [172,173]. Pulmozyme has low toxicity, so it has been tried repeatedly to relieve exacerbations in patients with cancer. The use of Pulmozyme in patients with head and neck cancer after radiochemotherapy reduced the viscosity of oropharyngeal secretions, which increased secondary indicators of quality of life [174]. The application of Pulmozyme makes it possible to cope with lung atelectasis, which is a frequent complication of lung cancer, in a safer manner than conservative treatment (therapeutic bronchoscopy) [175]. Pulmozyme inhalation also decreased excessive NET density in lungs, which reduced the risk of cancer-associated thrombosis [176]. However, there are still no studies devoted to the direct use of Pulmozyme or other dornase compositions for the treatment of oncological diseases.

A method for treating tumors by using bpDNase I and human recombinant DNase I was patented in 2003 by Genkin and colleagues [177] and had been supplemented by various patents up to 2015 [178]; however, the therapy using DNases has not become widespread. Summarizing all the above, DNase I can be suggested as a new effective drug for the treatment of diseases associated with the excessive release of pathological cfDNA, NETs as well as viral and bacterial DNA, and it is a promising anti-metastatic drug for a wide range of tumors.

## 9. Conclusions

Excess cfDNA is a dangerous factor in the development of various pathological states and malignant diseases. By themselves, cfDNA can activate acute inflammation, which negatively affects the general condition of the patient. cfDNA can be functionally dangerous: the involvement of cfDNA in the horizontal transfer of some ‘tumorigenic’ properties to normal cells—although it remains a controversial concept—could explain many of the inconsistencies in the data on metastasis development and reveal many potential targets for therapy. Excess cfDNA in NETs can also be dangerous and requires further study. DNases, both intracellular and secreted, constitute the main system by which tumor-derived potentially dangerous DNA is removed from an organism. Normal functioning of these DNases provides maintenance of cellular DNA homeostasis, and changes in their activity lead to tumor development. To date, the most promising strategy is to maintain the level of exDNases in the blood by using periodic administration of DNases or target delivery by vectors containing cellular DNases or their activators. In conclusion, DNases are novel, effective therapeutics for diseases associated with excess DNA, including excess circulating cfDNA, NET-integrated cfDNA as well as viral and bacterial DNA. This strategy could be promising for a wide range of tumors.

## Figures and Tables

**Table 1:** DNases providing intracellular and extracellular catabolism of DNA.

Name	Source ^1^	Location	Specificity ^2^	Cleavage Products ^3^	Ion Dependency	Optimal pH	Structure	Details
Intracellular catabolism of DNA								
**DNase IL1** **(DNase X)**	All cells	Endoplasmic reticulum, extracellular membrane	Chromatin	3′OH, 5′P oligonucleosomes with ds-breaks [127]	Ca^2+^, Mg^2+^	6–8	Capable of homodimerization [128]	C-terminal signal peptide, an N-linked glycosylation site and C-terminal hydrophobic domain; inactivated with Zn^2+^ and Apo10
**DNA fragmentation factor B (DFFB, DFF40 or CAD)**	All cells	Endoplasmic reticulum and nucleus	5′A(G)→3′X >> 5′C(T)→3′X chromatin and naked DNA [127]	3′OH, 5′P oligonucleosomes with ds-breaks [127]	Mg^2+^	6–8	Heterodimer	Usually bound with inhibitor DFFA; activated by cleavage of inhibitor with caspase-3
**Endonuclease G (EndoG)**	All cells	Mitochondria, migrates to the nucleus under apoptosis	poly(dG), poly(dC) >> others; ssDNA and dsDNA in chromatin; DNA/RNA heteroduplexes [129]	3′OH, 5′P oligonucleosomes with ds-breaks and internal ss- nicks [129]	Mg^2+^/Mn^2+^	Biphasic pH optima: 9 and 7 [129]	Homodimer	ββα-Me-finger; normally bound by Hsp70 and CHIP; inactivated with Fe^2+^ and Zn^2+^ [129]
**Extracellular catabolism of cfDNA**								
**DNase I**	Predominantly expressed in exocrine cells in the gastrointestinal tract, salivary glands, and kidneys; endothelial cells	Extracellular space	5′-T > C >> A,G→3′X; naked dsDNA >> ssDNA; DNA in DNA/RNA heteroduplexes; slight efficacy to chromatin [127]	3′OH,5′P mononucleosomes and tetranucleotides [127]	Ca^2+^, Mg^2+^	6–8	Monomer	Inactivated with Zn^2+^ and G-actin
**DNase IL3** **(DNAse γ)**	Predominantly expressed in the liver and spleen; endothelial cells; macrophages and dendritic cells	Endoplasmic reticulum, nucleus and extracellular space	5′ C > T >> A, G→3′X; chromatin DNA in lipid–membrane particles [130]	3′OH, 5′P mononucleosomes [127]	Ca^2+^, Mg^2+^	6–8		Inactivated with Zn^2+^ and heparin; it has a positively charged C-terminal sequence allowing transfer to the nucleus and encapsulation in MVs

^1^ Source includes only normal cells according to GenBank and Uniprot data, but not tumor cells. ^2^ X–any nucleotide. ^3^ P–phosphate group. Abbreviations: dsDNA, double-stranded DNA; ssDNA, single-stranded DNA.

## References

[1] Mandel P., Metais P. (1948). Les acides nucléiques du plasma sanguin chez l’homme. CR Seances Soc. Biol. Fil..

[2] Stroun M., Anker P., Maurice P., Gahan P.B. (1977). Circulating Nucleic Acids in Higher Organisms. Int. Rev. Cytol..

[3] Corcoran R.B., Chabner B.A. (2018). Application of Cell-free DNA Analysis to Cancer Treatment. N. Engl. J. Med..

[4] Bianchi D.W., Chiu R.W. (2018). Sequencing of Circulating Cell-free DNA during Pregnancy. N. Engl. J. Med..

[5] Dar P., Shani H., Evans M.I. (2016). Cell-free DNA: Comparison of technologies. Clin. Lab. Med..

[6] Lee K.-H., Shin T.-J., Kim W.-H., Lee S.Y., Cho J.-Y. (2019). Methylation of LINE-1 in cell-free DNA serves as a liquid biopsy biomarker for human breast cancers and dog mammary tumors. Sci. Rep..

[7] Ulrich B.C., Paweletz C.P. (2018). Cell-Free DNA in Oncology: Gearing up for Clinic. Ann. Lab. Med..

[8] Oellerich M., Schütz E., Beck J., Kanzow P., Plowman P.N., Weiss G.J., Walson P.D. (2017). Using circulating cell-free DNA to monitor personalized cancer therapy. Crit. Rev. Clin. Lab. Sci..

[9] Bedin C., Enzo M.V., Del Bianco P., Pucciarelli S., Nitti D., Agostini M. (2016). Diagnostic and prognostic role of cell-free DNA testing for colorectal cancer patients. Int. J. Cancer.

[10] (1999). Tumor DNA circulating in the plasma might play a role in metastasis. The hypothesis of the genometastasis. Histol. Histopathol..

[11] García-Olmo D.C., Domínguez C., García-Arranz M., Anker P., Stroun M., García-Verdugo J.M., García-Olmo D. (2010). Cell-Free Nucleic Acids Circulating in the Plasma of Colorectal Cancer Patients Induce the Oncogenic Transformation of Susceptible Cultured Cells. Cancer Res..

[12] Trejo-Becerril C., Pérez-Cárdenas E., Taja-Chayeb L., Anker P., Herrera-Goepfert R., Medina-Velázquez L.A., Hidalgo-Miranda A., Montiel M.D.P., Chávez-Blanco A., Cruz-Velázquez J. (2012). Cancer Progression Mediated by Horizontal Gene Transfer in an In Vivo Model. PLoS ONE.

[13] Bergsmedh A., Szeles A., Henriksson M., Bratt A., Folkman M.J., Spetz A.-L., Holmgren L. (2001). Horizontal transfer of oncogenes by uptake of apoptotic bodies. Proc. Natl. Acad. Sci. USA.

[14] Gaiffe E., Prétet J.-L., Launay S., Jacquin E., Saunier M., Hetzel G., Oudet P., Mougin C. (2012). Apoptotic HPV Positive Cancer Cells Exhibit Transforming Properties. PLoS ONE.

[15] Beyer C., Pisetsky D.S. (2009). The role of microparticles in the pathogenesis of rheumatic diseases. Nat. Rev. Rheumatol..

[16] Takei H., Araki A., Watanabe H., Ichinose A., Sendo F. (1996). Rapid killing of human neutrophils by the potent activator phorbol 12-myristate 13-acetate (PMA) accompanied by changes different from typical apoptosis or necrosis. J. Leukoc. Biol..

[17] Wartha F., Henriques-Normark B. (2008). ETosis: A Novel Cell Death Pathway. Sci. Signal..

[18] Chen Q., Zhang L., Li X., Zhuo W. (2021). Neutrophil Extracellular Traps in Tumor Metastasis: Pathological Functions and Clinical Applications. Cancers.

[19] Lake J.A., Jain R., Rivera M.C., Sinelnikov Y.D., Chen G., Neuville D.R., Vaughan M.T., Liebermann R.C. (1999). Mix and Match in the Tree of Life. Science.

[20] Ochman H., Lawrence J.G., Groisman E.A. (2000). Lateral gene transfer and the nature of bacterial innovation. Nature.

[21] Stegemann S., Bock R. (2009). Exchange of Genetic Material Between Cells in Plant Tissue Grafts. Science.

[22] Bhargava P.M., Shanmugam G. (1971). Uptake of Nonviral Nucleic Acids by Mammalian Cells. Prog. Nucleic Acid Res. Mol. Biol..

[23] Gahan P.B., Stroun M., Kikuchi Y., Rykova E. (2012). The biology of circulating nucleic acids in plasma and serum (CNAPS). Extracellular Nucleic Acids: Nucleic Acids and Molecular Biology.

[24] Karpfel Z., Šlotová J., Paleček E. (1963). Chromosome aberrations produced by deoxyribonucleic acids in mice. Exp. Cell Res..

[25] Szybalska E.H., Szybalski W. (1962). Genetics of human cell lines, iv. dna-mediated heritable transformation of a biochemical trait. Proc. Natl. Acad. Sci. USA.

[26] Stroun M., Anker P. (1972). Nucleic acids spontaneously released by living frog auricles. Biochem. J..

[27] Stroun M., Anker P. (1972). In vitro synthesis of DNA spontaneously released by bacteria or frog auricles. Biochimie.

[28] Gahan P.B., Stroun M. (2010). The virtosome-a novel cytosolic informative entity and intercellular messenger. Cell Biochem. Funct..

[29] Leon S.A., Shapiro B., Sklaroff D.M., Yaros M.J. (1977). Free DNA in the serum of cancer patients and the effect of therapy. Cancer Res..

[30] Boddy J.L., Gal S., Malone P.R., Harris A., Wainscoat J.S. (2005). Prospective Study of Quantitation of Plasma DNA Levels in the Diagnosis of Malignant versus Benign Prostate Disease. Clin. Cancer Res..

[31] Wimberger P., Roth C., Pantel K., Kasimir-Bauer S., Kimmig R., Schwarzenbach H. (2010). Impact of platinum-based chemotherapy on circulating nucleic acid levels, protease activities in blood and disseminated tumor cells in bone marrow of ovarian cancer patients. Int. J. Cancer.

[32] Kamat A.A., Bs M.B., Urbauer D.L., Dang D., Han L.Y., Godwin A.K., Karlan B.Y., Simpson J.L., Gershenson D.M., Coleman R.L. (2010). Plasma cell-free DNA in ovarian cancer: An independent prognostic biomarker. Cancer.

[33] Sunami E., Vu A.-T., Nguyen S.L., Giuliano A.E., Hoon D.S.B. (2008). Quantification of LINE1 in Circulating DNA as a Molecular Biomarker of Breast Cancer. Ann. N. Y. Acad. Sci..

[34] Allen D., Butt A., Cahill D., Wheeler M., Popert R., Swaminathan R. (2004). Role of Cell-Free Plasma DNA as a Diagnostic Marker for Prostate Cancer. Ann. N. Y. Acad. Sci..

[35] Schwarzenbach H., Stoehlmacher J., Pantel K., Goekkurt E. (2008). Detection and Monitoring of Cell-Free DNA in Blood of Patients with Colorectal Cancer. Ann. N. Y. Acad. Sci..

[36] Chun F.K.-H., Müller I., Lange I., Friedrich M.G., Erbersdobler A., Karakiewicz P.I., Graefen M., Pantel K., Huland H., Schwarzenbach H. (2006). Circulating tumour-associated plasma DNA represents an independent and informative predictor of prostate cancer. BJU Int..

[37] Tran N.H., Kisiel J., Roberts L.R. (2021). Using cell-free DNA for HCC surveillance and prognosis. JHEP Rep..

[38] Russo A., Incorvaia L., Del Re M., Malapelle U., Capoluongo E., Gristina V., Castiglia M., Danesi R., Fassan M., Giuffrè G. (2021). The molecular profiling of solid tumors by liquid biopsy: A position paper of the AIOM–SIAPEC-IAP–SIBioC–SIC–SIF Italian Scientific Societies. ESMO Open.

[39] Fleischhacker M., Schmidt B. (2007). Circulating nucleic acids (CNAs) and cancer—A survey. Biochim. Biophys. Acta Rev. Cancer.

[40] Jerónimo C., Nomoto S., Caballero O.L., Usadel H., Henrique R., Varzim G., Oliveira J., Lopes C., Fliss M.S., Sidransky D. (2001). Mitochondrial mutations in early stage prostate cancer and bodily fluids. Oncogene.

[41] Nomoto S., Yamashita K., Koshikawa K., Nakao A., Sidransky D. (2002). Mitochondrial D-loop mutations as clonal markers in multicentric hepatocellular carcinoma and plasma. Clin. Cancer Res..

[42] Belancio V.P., Engel A., Deininger P.L. (2010). All y’all need to know ‘bout retroelements in cancer. Semin. Cancer Biol..

[43] Carreira P., Richardson S., Faulkner G.J. (2013). L1 retrotransposons, cancer stem cells and oncogenesis. FEBS J..

[44] Kemp J.R., Longworth M.S. (2015). Crossing the LINE Toward Genomic Instability: LINE-1 Retrotransposition in Cancer. Front. Chem..

[45] Frickhofen N., Müller E., Sandherr M., Binder T., Bangerter M., Wiest C., Enz M., Heimpel H. (1997). Rearranged Ig Heavy Chain DNA Is Detectable in Cell-Free Blood Samples of Patients With B-Cell Neoplasia. Blood.

[46] Cirmena G., Dameri M., Ravera F., Fregatti P., Ballestrero A., Zoppoli G. (2021). Assessment of Circulating Nucleic Acids in Cancer: From Current Status to Future Perspectives and Potential Clinical Applications. Cancers.

[47] Rhodes C.H., Honsinger C., Sorenson G.D. (1994). Detection of Tumor-derived DNA in Cerebrospinal Fluid. J. Neuropathol. Exp. Neurol..

[48] Combaret V., Audoynaud C., Iacono I., Favrot M.-C., Schell M., Bergeron C., Puisieux A. (2002). Circulating MYCN DNA as a tumor-specific marker in neuroblastoma patients. Cancer Res..

[49] Gotoh T., Hosoi H., Iehara T., Kuwahara Y., Osone S., Tsuchiya K., Ohira M., Nakagawara A., Kuroda H., Sugimoto T. (2005). Prediction ofMYCNAmplification in Neuroblastoma Using Serum DNA and Real-Time Quantitative Polymerase Chain Reaction. J. Clin. Oncol..

[50] Beck J., Urnovitz H.B., Riggert J., Clerici M., Schütz E. (2009). Profile of the Circulating DNA in Apparently Healthy Individuals. Clin. Chem..

[51] van der Vaart M., Semenov D.V., Kuligina E., Richter V.A., Pretorius P.J. (2009). Characterisation of circulating DNA by parallel tagged sequencing on the 454 platform. Clin. Chim. Acta.

[52] Fan H.C., Blumenfeld Y.J., Chitkara U., Hudgins L., Quake S.R. (2008). Noninvasive diagnosis of fetal aneuploidy by shotgun sequencing DNA from maternal blood. Proc. Natl. Acad. Sci. USA.

[53] Batzer M.A., Deininger P.L. (2002). Alu repeats and human genomic diversity. Nat. Rev. Genet..

[54] Akers S.N., Moysich K., Zhang W., Lai G.C., Miller A., Lele S., Odunsi K., Karpf A.R. (2014). LINE1 and Alu repetitive element DNA methylation in tumors and white blood cells from epithelial ovarian cancer patients. Gynecol. Oncol..

[55] Sikora K., Bedin C., Vicentini C., Malpeli G., D’Angelo E., Sperandio N., Lawlor R.T., Bassi C., Tortora G., Nitti D. (2015). Evaluation of cell-free DNA as a biomarker for pancreatic malignancies. Int. J. Biol. Markers.

[56] Lehner J., Stötzer O.J., Fersching D., Nagel D., Holdenrieder S. (2013). Circulating plasma DNA and DNA integrity in breast cancer patients undergoing neoadjuvant chemotherapy. Clin. Chim. Acta.

[57] Iskow R.C., McCabe M.T., Mills R., Torene S., Pittard W.S., Neuwald A.F., Van Meir E.G., Vertino P.M., Devine S.E. (2010). Natural Mutagenesis of Human Genomes by Endogenous Retrotransposons. Cell.

[58] Duforestel M., Briand J., Bougras-Cartron G., Heymann D., Frenel J.-S., Vallette F.M., Cartron P.-F. (2020). Cell-free circulating epimarks in cancer monitoring. Epigenomics.

[59] Hur J., Lee K. (2021). Characteristics and Clinical Application of Extracellular Vesicle-Derived DNA. Cancers.

[60] Helman E., Lawrence M.S., Stewart C., Sougnez C., Getz G., Meyerson M. (2014). Somatic retrotransposition in human cancer revealed by whole-genome and exome sequencing. Genome Res..

[61] Rodríguez-Martín C., Cidre F., Fernández-Teijeiro A., Gómez-Mariano G., De La Vega L., Ramos P., Zaballos A., Monzón S., Alonso J. (2016). Familial retinoblastoma due to intronic LINE-1 insertion causes aberrant and noncanonical mRNA splicing of the RB1 gene. J. Hum. Genet..

[62] Stacey S.N., Kehr B., Gudmundsson J., Zink F., Jonasdottir A., Gudjonsson S.A., Sigurdsson A., Halldorsson B.V., Agnarsson B.A., Benediktsdottir K.R. (2016). Insertion of an SVA-E retrotransposon into theCASP8gene is associated with protection against prostate cancer. Hum. Mol. Genet..

[63] Alekseeva L.A., Sen’Kova A.V., Zenkova M.A., Mironova N.L. (2020). Targeting Circulating SINEs and LINEs with DNase I Provides Metastases Inhibition in Experimental Tumor Models. Mol. Ther.-Nucleic Acids.

[64] Clark S.R., Ma A.C., Tavener S.A., McDonald B., Goodarzi Z., Kelly M.M., Patel K.D., Chakrabarti S., McAvoy E., Sinclair G.D. (2007). Platelet TLR4 activates neutrophil extracellular traps to ensnare bacteria in septic blood. Nat. Med..

[65] Roshan M.H.K., Tambo A., Pace N.P. (2016). The Role of TLR2, TLR4, and TLR9 in the Pathogenesis of Atherosclerosis. Int. J. Inflamm..

[66] Yasuda K., Richez C., Uccellini M., Richards R.J., Bonegio R., Akira S., Monestier M., Corley R., Viglianti G., Marshak-Rothstein A. (2009). Requirement for DNA CpG Content in TLR9-Dependent Dendritic Cell Activation Induced by DNA-Containing Immune Complexes. J. Immunol..

[67] Liu R., Zhao E., Wang F., Cui H. (2020). CCDC25: Precise navigator for neutrophil extracellular traps on the prometastatic road. Signal Transduct. Target. Ther..

[68] Yang L., Liu Q., Zhang X., Liu X., Zhou B., Chen J., Huang D., Li J., Li H., Chen F. (2020). DNA of neutrophil extracellular traps promotes cancer metastasis via CCDC25. Nature.

[69] Budker V., Godovikov A., Naumova L., Slepneva I. (1980). Interaction of polynucleotides with natural and model membranes. Nucleic Acids Res..

[70] Bryzgunova O., Laktionov P. (2015). Generation of blood circulating DNA: The sources, peculiarities of circulation and structure. Biomeditsinskaya Khimiya.

[71] Kontouzov S., Cabrespines A., Amoura Z., Chabre H., Lotton C., Bach J.-F. (1996). Binding of nucleosomes to a cell surface receptor: Redistribution and endocytosis in the presence of lupus antibodies. Eur. J. Immunol..

[72] Hefeneider S.H., Cornell K.A., Brown L.E., Bakke A.C., McCoy S.L., Bennett R.M. (1992). Nucleosomes and DNA bind to specific cell-surface molecules on murine cells and induce cytokine production. Clin. Immunol. Immunopathol..

[73] Watson K., Gooderham N., Davies D.S., Edwards R.J. (1999). Nucleosomes Bind to Cell Surface Proteoglycans. J. Biol. Chem..

[74] Kubota T., Kanai Y., Miyasaka N. (1990). Interpretation of the cross-reactivity of anti-DNA antibodies with cell surface proteins: The role of cell surface histones. Immunol. Lett..

[75] Atluri S., Ragkousi K., Cortezzo D.E., Setlow P. (2006). Cooperativity Between Different Nutrient Receptors in Germination of Spores of Bacillus subtilis and Reduction of This Cooperativity by Alterations in the GerB Receptor. J. Bacteriol..

[76] Marsman G., Zeerleder S., Luken B.M. (2016). Extracellular histones, cell-free DNA, or nucleosomes: Differences in immunostimulation. Cell Death Dis..

[77] Cai J., Han Y., Ren H., Chen C., He D., Zhou L., Eisner G.M., Asico L.D., Jose P.A., Zeng C. (2013). Extracellular vesicle-mediated transfer of donor genomic DNA to recipient cells is a novel mechanism for genetic influence between cells. J. Mol. Cell Biol..

[78] Thakur B.K., Zhang H., Becker A., Matei I., Huang Y., Costa-Silva B., Zheng Y., Hoshino A., Brazier H., Xiang J. (2014). Double-stranded DNA in exosomes: A novel biomarker in cancer detection. Cell Res..

[79] Fernando M.R., Jiang C., Krzyzanowski G.D., Ryan W.L. (2017). New evidence that a large proportion of human blood plasma cell-free DNA is localized in exosomes. PLoS ONE.

[80] Jeppesen D.K., Fenix A.M., Franklin J.L., Higginbotham J.N., Zhang Q., Zimmerman L.J., Liebler D.C., Ping J., Liu Q., Evans R. (2019). Reassessment of Exosome Composition. Cell.

[81] Msc E.L., Sanz-García A., Visakorpi T., Escobedo-Lucea C., Siljander P., Ayuso-Sacido A., Yliperttula M. (2014). Different gDNA content in the subpopulations of prostate cancer extracellular vesicles: Apoptotic bodies, microvesicles, and exosomes. Prostate.

[82] Lee T.H., Chennakrishnaiah S., Audemard E., Montermini L., Meehan B., Rak J. (2014). Oncogenic ras-driven cancer cell vesiculation leads to emission of double-stranded DNA capable of interacting with target cells. Biochem. Biophys. Res. Commun..

[83] Cai J., Wu G., Tan X., Han Y., Chen C., Li C., Wang N., Zou X., Chen X., Zhou F. (2014). Transferred BCR/ABL DNA from K562 Extracellular Vesicles Causes Chronic Myeloid Leukemia in Immunodeficient Mice. PLoS ONE.

[84] Pilsczek F.H., Salina D., Poon K.K.H., Fahey C., Yipp B.G., Sibley C.D., Robbins S.M., Green F.H.Y., Surette M.G., Sugai M. (2010). A Novel Mechanism of Rapid Nuclear Neutrophil Extracellular Trap Formation in Response toStaphylococcus aureus. J. Immunol..

[85] Yipp B.G., Petri B., Salina D., Jenne C.N., Scott B.N.V., Zbytnuik L.D., Pittman K., Asaduzzaman M., Wu K., Meijndert H.C. (2012). Infection-induced NETosis is a dynamic process involving neutrophil multitasking in vivo. Nat. Med..

[86] Brinkmann V., Reichard U., Goosmann C., Fauler B., Uhlemann Y., Weiss D.S., Weinrauch Y., Zychlinsky A. (2004). Neutrophil extracellular traps kill bacteria. Science.

[87] Kobayashi S.D., Braughton K.R., Whitney A.R., Voyich J.M., Schwan T.G., Musser J.M., DeLeo F.R. (2003). Bacterial pathogens modulate an apoptosis differentiation program in human neutrophils. Proc. Natl. Acad. Sci. USA.

[88] Liu Y., Liu L. (2019). The pro-tumor effect and the anti-tumor effect of neutrophils extracellular traps. Biosci. Trends.

[89] Demers M., Wagner D.D. (2014). NETosis: A New Factor in Tumor Progression and Cancer-Associated Thrombosis. Semin. Thromb. Hemost..

[90] Jorch S.K., Kubes P. (2017). An emerging role for neutrophil extracellular traps in noninfectious disease. Nat. Med..

[91] Gregory A.D., Houghton A.M. (2011). Tumor-Associated Neutrophils: New Targets for Cancer Therapy: Figure 1. Cancer Res..

[92] Ho-Tin-Noé B., Carbo C., Demers M., Cifuni S.M., Goerge T., Wagner D.D. (2009). Innate Immune Cells Induce Hemorrhage in Tumors during Thrombocytopenia. Am. J. Pathol..

[93] Berger-Achituv S., Brinkmann V., Abu Abed U., Kühn L.I., Ben-Ezra J., Elhasid R., Zychlinsky A. (2013). A proposed role for neutrophil extracellular traps in cancer immunoediting. Front. Immunol..

[94] Guglietta S., Chiavelli A., Zagato E., Krieg C., Gandini S., Ravenda P.S., Bazolli B., Lu B., Penna G., Rescigno M. (2016). Coagulation induced by C3aR-dependent NETosis drives protumorigenic neutrophils during small intestinal tumorigenesis. Nat. Commun..

[95] Tohme S., Yazdani H.O., Al-Khafaji A.B., Chidi A.P., Loughran P., Mowen K.A., Wang Y., Simmons R.L., Huang H., Tsung A. (2016). Neutrophil extracellular traps promote the development and progression of liver metastases after surgical stress. Cancer Res..

[96] Demers M., Krause D.S., Schatzberg D., Martinod K., Voorhees J.R., Fuchs T.A., Scadden D.T., Wagner D.D. (2012). Cancers predispose neutrophils to release extracellular DNA traps that contribute to cancer-associated thrombosis. Proc. Natl. Acad. Sci. USA.

[97] Najmeh S., Cools-Lartigue J., Rayes R.F., Gowing S., Vourtzoumis P., Bourdeau F., Giannias B., Berube J., Rousseau S., Ferri L.E. (2017). Neutrophil extracellular traps sequester circulating tumor cells via β1-integrin mediated interactions. Int. J. Cancer.

[98] Zhu T., Zou X., Yang C., Li L., Wang B., Li R., Li H., Xu Z., Huang D., Wu Q. (2021). Neutrophil extracellular traps promote gastric cancer metastasis by inducing epithelial-mesenchymal transition. Int. J. Mol. Med..

[99] Rayes R.F., Mouhanna J.G., Nicolau I., Bourdeau F., Giannias B., Rousseau S., Quail D., Walsh L., Sangwan V., Bertos N. (2019). Primary tumors induce neutrophil extracellular traps with targetable metastasis-promoting effects. JCI Insight.

[100] Arelaki S., Arampatzioglou A., Kambas K., Papagoras C., Miltiades P., Angelidou I., Mitsios A., Kotsianidis I., Skendros P., Sivridis E. (2016). Gradient Infiltration of Neutrophil Extracellular Traps in Colon Cancer and Evidence for Their Involvement in Tumour Growth. PLoS ONE.

[101] Schedel F., Mayer-Hain S., Pappelbaum K.I., Metze D., Stock M., Goerge T., Loser K., Sunderkötter C., Luger T.A., Weishaupt C. (2019). Evidence and impact of neutrophil extracellular traps in malignant melanoma. Pigment. Cell Melanoma Res..

[102] Breitbach C.J., De Silva N.S., Falls T., Aladl U., Evgin L., Paterson J., Sun Y.Y., Roy D., Rintoul J.L., Daneshmand M. (2011). Targeting Tumor Vasculature with an Oncolytic Virus. Mol. Ther..

[103] Park J., Wysocki R.W., Amoozgar Z., Maiorino L., Fein M.R., Jorns J., Schott A.F., Kinugasa-Katayama Y., Lee Y., Won N.H. (2016). Cancer cells induce metastasis-supporting neutrophil extracellular DNA traps. Sci. Transl. Med..

[104] Jin W., Xu H.-X., Zhang S.-R., Li H., Wang W.-Q., Gao H.-L., Wu C.-T., Xu J.-Z., Qi Z.-H., Li S. (2018). Tumor-Infiltrating NETs Predict Postsurgical Survival in Patients with Pancreatic Ductal Adenocarcinoma. Ann. Surg. Oncol..

[105] McInturff A.M., Cody M.J., Elliott E.A., Glenn J.W., Rowley J.W., Rondina M.T., Yost C.C. (2012). Mammalian target of rapamycin regulates neutrophil extracellular trap formation via induction of hypoxia-inducible factor 1 α. Blood.

[106] Cools-Lartigue J., Spicer J., McDonald B., Gowing S., Chow S., Giannias B., Bourdeau F., Kubes P., Ferri L. (2013). Neutrophil extracellular traps sequester circulating tumor cells and promote metastasis. J. Clin. Investig..

[107] Gould T.J., Vu T.T., Swystun L.L., Dwivedi D.J., Mai S.H., Weitz J.I., Liaw P.C. (2014). Neutrophil extracellular traps promote thrombin generation through platelet-dependent and platelet-independent mechanisms. Arter. Thromb. Vasc. Biol..

[108] Von Brühl M.L., Stark K., Steinhart A., Chandraratne S., Konrad I., Lorenz M., Khandoga A., Tirniceriu A., Coletti R., Köllnberger M. (2012). Monocytes, neutrophils, and platelets cooperate to initiate and propagate venous thrombosis in mice in vivo. J. Exp. Med..

[109] Kambas K., Chrysanthopoulou A., Vassilopoulos D., Apostolidou E., Skendros P., Girod A., Arelaki S., Froudarakis M., Nakopoulou L., Giatromanolaki A. (2013). Tissue factor expression in neutrophil extracellular traps and neutrophil derived microparticles in antineutrophil cytoplasmic antibody associated vasculitis may promote thromboinflammation and the thrombophilic state associated with the disease. Ann. Rheum. Dis..

[110] Kambas K., Mitroulis I., Apostolidou E., Girod A., Chrysanthopoulou A., Pneumatikos I., Skendros P., Kourtzelis I., Koffa M., Kotsianidis I. (2012). Autophagy Mediates the Delivery of Thrombogenic Tissue Factor to Neutrophil Extracellular Traps in Human Sepsis. PLoS ONE.

[111] Cedervall J., Zhang Y., Huang H., Zhang L., Femel J., Dimberg A., Olsson A.-K. (2015). Neutrophil Extracellular Traps Accumulate in Peripheral Blood Vessels and Compromise Organ Function in Tumor-Bearing Animals. Cancer Res..

[112] Janus N., Launay-Vacher V., Byloos E., Machiels J.P., Duck L., Kerger J., Wynendaele W., Canon J.-L., Lybaert W., Nortier J. (2010). Cancer and renal insufficiency results of the BIRMA study. Br. J. Cancer.

[113] Launay-Vacher V. (2010). Epidemiology of Chronic Kidney Disease in Cancer Patients: Lessons from the IRMA Study Group. Semin. Nephrol..

[114] Launay-Vacher V., Oudard S., Janus N., Gligorov J., Pourrat X., Rixe O., Morere J.-F., Beuzeboc P., Deray G. (2007). Prevalence of Renal Insufficiency in cancer patients and implications for anticancer drug management: The renal insufficiency and anticancer medications (IRMA) study. Cancer.

[115] Thålin C., Demers M., Blomgren B., Wong S.L., von Arbin M., von Heijne A., Laska A.C., Wallén H., Wagner D.D., Aspberg S. (2016). NETosis promotes cancer-associated arterial microthrombosis presenting as ischemic stroke with troponin elevation. Thromb. Res..

[116] Yu S.C., Lee S.W., Jiang P., Leung T.Y., Chan K.A., Chiu R.W., Lo Y.D. (2013). High-Resolution Profiling of Fetal DNA Clearance from Maternal Plasma by Massively Parallel Sequencing. Clin. Chem..

[117] Han D.S., Lo Y.D. (2021). The Nexus of cfDNA and Nuclease Biology. Trends Genet..

[118] Coy J.F., Velhagen I., Himmele R., Delius H., Poustka A., Zentgraf H. (1996). Isolation, differential splicing and protein expression of a DNase on the human X chromosome. Cell Death Differ..

[119] Shiokawa D., Matsushita T., Shika Y., Shimizu M., Maeda M., Tanuma S.-I. (2007). DNase X Is a Glycosylphosphatidylinositol-anchored Membrane Enzyme That Provides a Barrier to Endocytosis-mediated Transfer of a Foreign Gene. J. Biol. Chem..

[120] Shiokawa D., Tanuma S.-I. (2000). Characterization of Human DNase I Family Endonucleases and Activation of DNase γ during Apoptosis. Biochemistry.

[121] Pal K., Zhao Y., Wang Y., Wang X. (2021). Ubiquitous membrane-bound DNase activity in podosomes and invadopodia. J. Cell Biol..

[122] Grimm M., Cetindis M., Lehmann M., Biegner T., Munz A., Teriete P., Reinert S. (2014). Apoptosis resistance-related ABCB5 and DNaseX (Apo10) expression in oral carcinogenesis. Acta Odontol. Scand..

[123] Taper H.S. (2008). Altered deoxyribonuclease activity in cancer cells and its role in non toxic adjuvant cancer therapy with mixed vitamins C and K3. Anticancer. Res..

[124] Grimm M., Schmitt S., Teriete P., Biegner T., Stenzl A., Hennenlotter J., Muhs H.-J., Munz A., Nadtotschi T., König K. (2013). A biomarker based detection and characterization of carcinomas exploiting two fundamental biophysical mechanisms in mammalian cells. BMC Cancer.

[125] Wang Y., Zhao Y., Sarkar A., Wang X. (2018). Optical sensor revealed abnormal nuclease spatial activity on cancer cell membrane. J. Biophotonics.

[126] Coy J.F. (2017). EDIM-TKTL1/Apo10 Blood Test: An Innate Immune System Based Liquid Biopsy for the Early Detection, Characterization and Targeted Treatment of Cancer. Int. J. Mol. Sci..

[127] Woo E.-J., Kim Y.-G., Kim M.-S., Han W.-D., Shin S., Robinson H., Park S.-Y., Oh B.-H. (2004). Structural Mechanism for Inactivation and Activation of CAD/DFF40 in the Apoptotic Pathway. Mol. Cell.

[128] Los M., Neubüser D., Coy J.F., Mozoluk M., Poustka A., Schulze-Osthoff K. (2000). Functional Characterization of DNase X, a Novel Endonuclease Expressed in Muscle Cells. Biochemistry.

[129] Widlak P., Li L.Y., Wang X., Garrard W.T. (2001). Action of Recombinant Human Apoptotic Endonuclease G on Naked DNA and Chromatin Substrates. J. Biol. Chem..

[130] Sisirak V., Sally B., D’Agati V., Martinez-Ortiz W., Özçakar Z., David J., Rashidfarrokhi A., Yeste A., Panea C., Chida A.S. (2016). Digestion of Chromatin in Apoptotic Cell Microparticles Prevents Autoimmunity. Cell.

[131] Miles M.A., Harris M.A., Hawkins C.J. (2019). Proteasome inhibitors trigger mutations via activation of caspases and CAD, but mutagenesis provoked by the HDAC inhibitors vorinostat and romidepsin is caspase/CAD-independent. Apoptosis.

[132] Gao Y., Zhang X., Wang T., Zhang Y., Wang Q., Hu Y. (2020). *HNRNPCL1*, *PRAMEF1*, *CFAP74*, and *DFFB*: Common Potential Biomarkers for Sporadic and Suspected Lynch Syndrome Endometrial Cancer. Cancer Manag. Res..

[133] Zhdanov D.D., Fahmi T., Wang X., Apostolov E.O., Sokolov N.N., Javadov S., Basnakian A.G. (2015). Regulation of Apoptotic Endonucleases by EndoG. DNA Cell Biol..

[134] Zhdanov D.D., Gladilina Y.A., Pokrovsky V.S., Grishin D.V., Grachev V., Orlova V., Pokrovskaya M.V., Alexandrova S.S., Plyasova A.A., Sokolov N.N. (2018). Endonuclease G modulates the alternative splicing of deoxyribonuclease 1 mRNA in human CD4+ T lymphocytes and prevents the progression of apoptosis. Biochimie.

[135] Barés G., Beà A., Hernández L., Navaridas R., Felip I., Megino C., Blasco N., Nadeu F., Campo E., Llovera M. (2021). ENDOG Impacts on Tumor Cell Proliferation and Tumor Prognosis in the Context of PI3K/PTEN Pathway Status. Cancers.

[136] Zhao M., Wang Y., Zhao Y., He S., Zhao R., Song Y., Cheng J., Gong Y., Xie J., Wang Y. (2020). Caspase-3 knockout attenuates radiation-induced tumor repopulation via impairing the ATM/p53/Cox-2/PGE2 pathway in non-small cell lung cancer. Aging.

[137] Hawes M.C., Curlango-Rivera G., Wen F., White G.J., VanEtten H.D., Xiong Z. (2011). Extracellular DNA: The tip of root defenses?. Plant Sci..

[138] Han D.S., Ni M., Chan R.W., Chan V.W., Lui K., Chiu R.W., Lo Y.D. (2020). The Biology of Cell-free DNA Fragmentation and the Roles of DNASE1, DNASE1L3, and DFFB. Am. J. Hum. Genet..

[139] Kishi K., Yasuda T., Takeshita H. (2001). DNase I: Structure, function, and use in medicine and forensic science. Leg. Med..

[140] Kochanek S., Renz D., Doerfler W. (1993). Differences in the accessibility of methylated and unmethylated DNA to DNase I. Nucleic Acids Res..

[141] Eulitz D., Mannherz H.G. (2007). Inhibition of deoxyribonuclease I by actin is to protect cells from premature cell death. Apoptosis.

[142] Rodriguez A.M., Rodina D., Nomuraa H., Morton C.C., Weremowiczb S., Schneider M.C. (1997). Identification, Localization, and Expression of Two Novel Human Genes Similar to Deoxyribonuclease I. Genomics.

[143] Wilber A., Lu M., Schneider M.C. (2002). Deoxyribonuclease I-like III Is an Inducible Macrophage Barrier to Liposomal Transfection. Mol. Ther..

[144] Napirei M., Wulf S., Eulitz D., Mannherz H.G., Kloeckl T. (2005). Comparative characterization of rat deoxyribonuclease 1 (Dnase1) and murine deoxyribonuclease 1-like 3 (Dnase1l3). Biochem. J..

[145] Mizuta R., Mizuta M., Araki S., Shiokawa D., Tanuma S.-I., Kitamura D. (2006). Action of apoptotic endonuclease DNase γ on naked DNA and chromatin substrates. Biochem. Biophys. Res. Commun..

[146] Jiménez-Alcázar M., Rangaswamy C., Panda R., Bitterling J., Simsek Y.J., Long A.T., Bilyy R., Krenn V., Renné C., Renné T. (2017). Host DNases prevent vascular occlusion by neutrophil extracellular traps. Science.

[147] Economidou-Karaoglou A., Lans M., Taper H.S., Michaux J.L., Roberfroid M. (1988). variations in serum alkaline dnase activity. A new means for therapeutic monitoring of malignant lymphomas. Cancer.

[148] Tamkovich S., Cherepanova A.V., Kolesnikova E.V., Rykova E.Y., Pyshnyi D.V., Vlassov V.V., Laktionov P.P. (2006). Circulating DNA and DNase Activity in Human Blood. Ann. N. Y. Acad. Sci..

[149] Cherepanova A.V., Tamkovich S.N., Vlassov V.V., Laktionov P.P. (2007). Blood deoxyribonuclease activity in health and diseases. Biochem. Suppl. Ser. B: Biomed. Chem..

[150] Golonka R.M., Yeoh B.S., Petrick J.L., Weinstein S.J., Albanes D., Gewirtz A.T., McGlynn K.A., Vijay-Kumar M. (2018). Deoxyribonuclease I Activity, Cell-Free DNA, and Risk of Liver Cancer in a Prospective Cohort. JNCI Cancer Spectr..

[151] Liu J., Yi J., Zhang Z., Cao D., Li L., Yao Y. (2021). Deoxyribonuclease 1-like 3 may be a potential prognostic biomarker associated with immune infiltration in colon cancer. Aging.

[152] Hazeldine J., Dinsdale R.J., Naumann D.N., Acharjee A., Bishop J.R.B., Lord J.M., Harrison P. (2021). Traumatic injury is associated with reduced deoxyribonuclease activity and dysregulation of the actin scavenging system. Burn. Trauma.

[153] Yasuda T., Kawai Y., Ueki M., Kishi K. (2005). Clinical applications of DNase I, a genetic marker already used for forensic identification. Leg. Med..

[154] De Lamirande G. (1961). Action of Deoxyribonuclease and Ribonuclease on the Growth of Ehrlich Ascites Carcinoma in Mice. Nature.

[155] Belyaeva M.I., Kyune M.F., Nuxhina A.M. (1964). Effect of bacterial deoxyribonuclease on Ehrlich’s ascities carcinoma cells in vitro. Fed. Proc. Transl. Suppl..

[156] Salganik R.I., Martynova R.P., Matienko N.A., Ronichevskaya G.M. (1967). Effect of Deoxyribonuclease on the Course of Lymphatic Leukaemia in AKR Mice. Nature.

[157] Sugihara S., Yamamoto T., Tanaka H., Kambara T., Hiraoka T., Miyauchi Y. (1993). Deoxyribonuclease treatment prevents blood-borne liver metastasis of cutaneously transplanted tumour cells in mice. Br. J. Cancer.

[158] Alcázar-Leyva S., Cerón E., Masso F., Montaño L.F., Gorocica P., Alvarado-Vásquez N. (2009). Incubation with DNase I inhibits tumor cell proliferation. Med Sci. Monit..

[159] Patutina O., Mironova N., Ryabchikova E., Popova N., Nikolin V., Kaledin V., Vlassov V., Zenkova M. (2011). Inhibition of metastasis development by daily administration of ultralow doses of RNase A and DNase I. Biochimie.

[160] Wen F., Shen A., Choi A., Gerner E.W., Shi J. (2013). Extracellular DNA in Pancreatic Cancer Promotes Cell Invasion and Metastasis. Cancer Res..

[161] Zou Y., Chen X., Xiao J., Zhou D.B., Lu X.X., Li W., Xie B., Kuang X., Chen Q. (2018). Neutrophil extracellular traps promote lipopolysaccharide-induced airway inflammation and mucus hypersecretion in mice. Oncotarget.

[162] Mohanty T., Fisher J., Bakochi A., Neumann A., Cardoso J.F.P., Karlsson C.A.Q., Pavan C., Lundgaard I., Nilson B., Reinstrup P. (2019). Neutrophil extracellular traps in the central nervous system hinder bacterial clearance during pneumococcal meningitis. Nat. Commun..

[163] Demkow U. (2021). Neutrophil Extracellular Traps (NETs) in Cancer Invasion, Evasion and Metastasis. Cancers.

[164] Spicer J.D., McDonald B., Cools-Lartigue J.J., Chow S.C., Giannias B., Kubes P., Ferri L.E. (2012). Neutrophils Promote Liver Metastasis via Mac-1–Mediated Interactions with Circulating Tumor Cells. Cancer Res..

[165] Hisada Y., Grover S.P., Maqsood A., Houston R., Ay C., Noubouossie D.F., Cooley B.C., Wallén H., Key N.S., Thålin C. (2019). Neutrophils and neutrophil extracellular traps enhance venous thrombosis in mice bearing human pancreatic tumors. Haematologica.

[166] Alekseeva L.A., Mironova N., Brenner E.V., Kurilshikov A., Patutina O.A., Zenkova M.A. (2017). Alteration of the exDNA profile in blood serum of LLC-bearing mice under the decrease of tumour invasion potential by bovine pancreatic DNase I treatment. PLoS ONE.

[167] Rosner K. (2011). DNase1: A new personalized therapy for cancer?. Expert Rev. Anticancer. Ther..

[168] Haig J.D.M. (2013). Eradication of Human Ovarian Cancer Cells by Transgenic Expression of Recombinant DNASE1, DNASE1L3, DNASE2, and DFFB Controlled by EGFR Promoter: Novel Strategy for Targeted Therapy of Cancer. J. Genet. Syndr. Gene Ther..

[169] Xia Y., He J., Zhang H., Wang H., Tetz G., Maguire C.A., Wang Y., Onuma A., Genkin D., Tetz V. (2020). AAV-mediated gene transfer of DNase I in the liver of mice with colorectal cancer reduces liver metastasis and restores local innate and adaptive immune response. Mol. Oncol..

[170] Yang L.-Y., Luo Q., Lu L., Zhu W.-W., Sun H.-T., Wei R., Lin Z.-F., Wang X.-Y., Wang C.-Q., Lu M. (2020). Increased neutrophil extracellular traps promote metastasis potential of hepatocellular carcinoma via provoking tumorous inflammatory response. J. Hematol. Oncol..

[171] Liu Y., Carmona-Rivera C., Moore E., Seto N.L., Knight J.S., Pryor M., Yang Z.-H., Hemmers S., Remaley A.T., Mowen K.A. (2018). Myeloid-Specific Deletion of Peptidylarginine Deiminase 4 Mitigates Atherosclerosis. Front. Immunol..

[172] Fuchs H.J., Borowitz D.S., Christiansen D.H., Morris E.M., Nash M.L., Ramsey B.W., Rosenstein B.J., Smith A.L., Wohl M.E. (1994). Effect of Aerosolized Recombinant Human DNase on Exacerbations of Respiratory Symptoms and on Pulmonary Function in Patients with Cystic Fibrosis. N. Engl. J. Med..

[173] Silverman R.A., Foley F., Dalipi R., Kline M., Lesser M. (2012). The use of rhDNAse in severely ill, non-intubated adult asthmatics refractory to bronchodilators: A pilot study. Respir. Med..

[174] Mittal B.B., Wang E., Sejpal S., Agulnik M., Mittal A., Harris J. (2013). Effect of Recombinant Human Deoxyribonuclease on Oropharyngeal Secretions in Patients with Head-and-Neck Cancers Treated with Radiochemotherapy. Int. J. Radiat. Oncol..

[175] Assallum H., Song T.Y., DeLorenzo L., Harris K. (2019). Bronchoscopic instillation of DNase to manage refractory lobar atelectasis in a lung cancer patient. Ann. Transl. Med..

[176] Várady C.B., Oliveira A.C., Monteiro R.Q., Gomes T. (2021). Recombinant human DNase I for the treatment of cancer-associated thrombosis: A pre-clinical study. Thromb. Res..

[177] Genkin D., Tets V., Tets G. (2005). Method for Treating Oncological Diseases. U.S. Patent.

[178] Genkin D., Tets G., Tets V. (2020). Method to Improve Safety and Efficacy of Anti-Cancer Therapy 2015. U.S. Patent.

